# 18F-Fluciclovine Uptake in an Incidentally Discovered Meningioma in a Patient With a History of Metastatic Prostate Cancer: A Case Report

**DOI:** 10.7759/cureus.46073

**Published:** 2023-09-27

**Authors:** Danielle A Touma, Horace Hayes, Francis J Cloran

**Affiliations:** 1 Radiology, Walter Reed National Military Medical Center, Bethesda, USA; 2 Radiology, Brooke Army Medical Center, San Antonio, USA

**Keywords:** retinoblastoma tumor suppressor gene mutation, pet-ct fluciclovine, axumin, prostate cancer, meningioma

## Abstract

Meningiomas are common, benign tumors found in the dural layers of the central nervous system (CNS) that are often found incidentally. ^18^F-fluciclovine is an amino acid radiotracer that is used to monitor the recurrence of prostate cancer due to its high uptake in prostate cancer cells. This case report outlines a patient with a retinoblastoma tumor suppressor gene (RB1) mutation and prostate cancer metastasis to the bone incidentally noted to have an enhancing, extra-axial mass on a screening MRI. On prior scans, the mass displayed increased ^18^F-fluciclovine uptake. Because prostate cancer can metastasize to the meninges, especially in older patients with advanced disease, consideration was given to the progression of his oncological disease. However, additional imaging validated the existence and size of the mass, making a meningioma the final diagnosis. Meningeal metastases can be virtually indistinguishable from other CNS tumors, including meningiomas appearing as single or multiple dural-based, enhancing masses, and without prior imaging, further investigation is warranted.

## Introduction

Meningiomas are common, benign tumors of the central nervous system that arise between dural layers. Symptomatology varies based on the size and location of the tumor and diagnosis is made using contrast-enhanced CT or MRI as well as assessment on longitudinal imaging. These tumors are often found incidentally for this reason. Fluorine 18 (^18^F) fluciclovine, or anti-1-amino-3-18F-fluorocyclobutane-1-carboxylic acid (FACBC), or Axumin, is an amino acid radiotracer that has high uptake in prostate cancer cells. This radiotracer is Food and Drug Administration (FDA)-approved to monitor the recurrence of prostate cancer. We describe a patient with a history of an RB1 mutation and prostate cancer with metastasis to the bone found to have an enhancing, extra-axial mass on a screening MRI. The mass demonstrated increased uptake of ^18^F-fluciclovine, and after further investigation, was determined to be a meningioma [1].

## Case presentation

**Figure 1:**
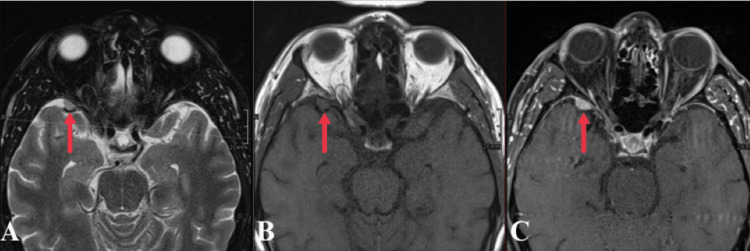
Axial T2 (A), T1 pre-contrast (B), and T1 post-contrast (C) MRI images demonstrate a T1/T2 isointense dural-based mass along the anterior middle cranial fossa measuring 10 x 8 x 10 mm with avid enhancement and dural tail

**Figure 2:**
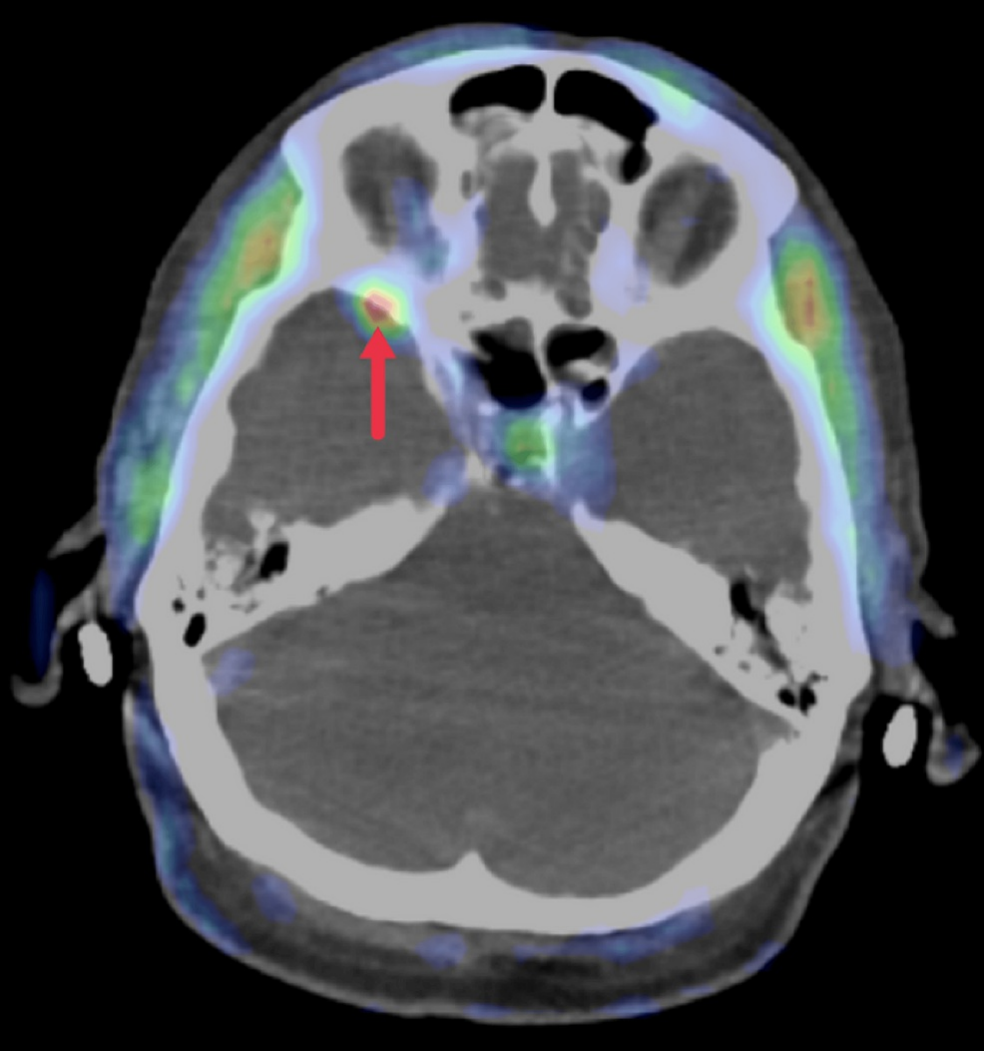
Axial 18F-fluciclovine PET/CT performed one year prior demonstrates increased uptake in the location of the described dural-based right middle cranial mass PET/CT: positron emission tomography/computed tomography

**Figure 3:**
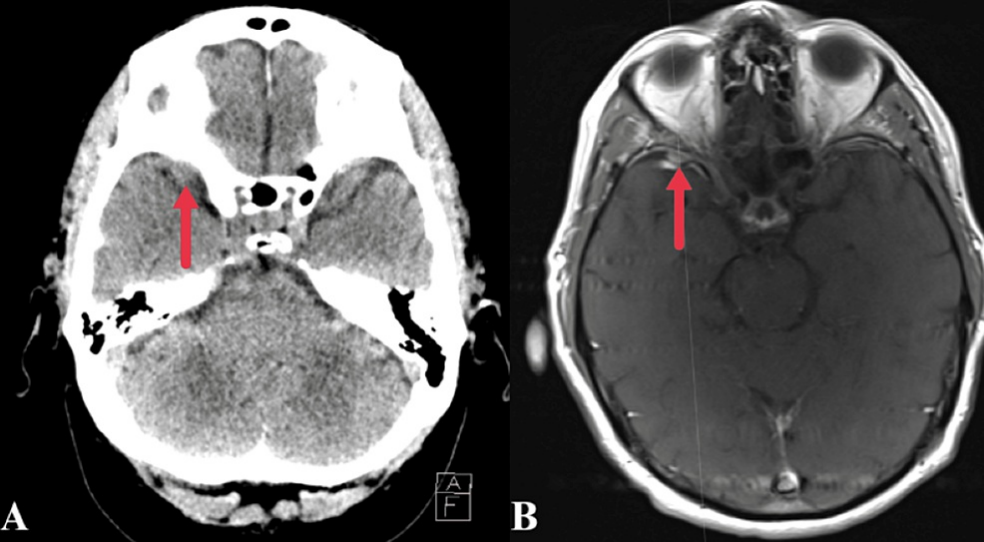
Axial CT (A) and T1 contrast-enhanced MRI (B) images performed two years prior demonstrate the same lesion, measuring 6 x 6 x 6 mm

The patient was a 64-year-old male with a history of RB1 mutation and high-risk castrate-naive prostate cancer status post robotic-assisted laparoscopic prostatectomy and radiotherapy complicated by subsequent oligometastatic disease to the left anterior superior iliac spine, also treated with radiotherapy. His current medications include abiraterone/prednisone, degarelix, and denosumab. Due to his RB1 mutation, he receives annual screening MRIs of the orbits. A recent MRI demonstrated an avidly enhancing, extra-axial, dural-based tumor along the right middle cranial fossa measuring up to 10 mm (Figures 1A-1C). The appearance was most consistent with a meningioma. However, considering the patient’s history and risk factors, metastatic disease was also a concern. Upon further review of the patient’s imaging, an ^18^F-fluciclovine PET/CT performed a year prior demonstrated an increased uptake in the middle cranial fossa, correlating with the mass on MRI and further raising suspicion (Figure 2). With continued probing, a CT and MRI of the brain from two years prior were found to have the same mass in the right middle cranial fossa, measuring up to 7 mm (Figures 3A, 3B). Given the indolent interval growth of the mass, meningioma was favored over metastatic disease secondary to prostate cancer or other RB1-related disease. Additionally, a low PSA and undetectable testosterone obtained one month prior to imaging make prostate cancer metastasis unlikely. Continued surveillance with MRI in three to six months was recommended given the history.

## Discussion

^18^F-fluciclovine is an analog of the amino acid leucine, which demonstrates high uptake in prostate cancer cells via amino acid transporters and is FDA-approved to monitor the recurrence of prostate cancer [2]. However, there are reports of increased ^18^F-fluciclovine uptake found incidentally in glial tumors and meningiomas [1]. In addition to the sodium-dependent amino acid transporter found in prostate cancer cells, other transporters, such as large neutral amino acid transporters, demonstrate uptake of ^18^F-fluciclovine. Although rare, advanced prostate cancer can metastasize to the meninges. One review study found that 90% of patients with meningeal metastasis had prior bone involvement, like in this patient, and the mean age of diagnosis was 63 [3]. Once meningeal metastasis develops, the mean time to death ranges from 2.3 to 9 months, depending on treatment [3]. While meningiomas are typically slow-growing, enhancing, extra-axial, dural-based tumors, meningeal metastases can be virtually indistinguishable appearing as single or multiple dural-based, enhancing masses [4]. In our patient, the diagnosis of meningioma was made based on a review of prior imaging. A CT and MRI of the brain performed two years prior demonstrated indolent growth, consistent with a meningioma.

In the absence of prior imaging, there are other routes to establish the diagnosis of meningioma over metastasis. Meningiomas may have restricted diffusion on DWI/ADC [5]. If the tumor can satisfactorily be imaged by spectroscopy, an alanine peak at 1.47 ppm is felt to be specific in the diagnosis of meningioma though can be difficult to identify within the spectrum and size as well as location may limit optimal spectrum acquisition [6]. Meningiomas can be visualized with somatostatin receptor (SSTR) ligands such as ^68^Ga-DOTATATE, DOTATOC, or DOTANOC, which can help confirm a suspected diagnosis [7]. The use of a more common radiotracer, FDG, is limited secondary to meningioma's slow growth pattern and low glucose metabolism [7]. FGD is not specific for tumor tissue and may have increased uptake in areas of inflammation. Furthermore, as the majority of meningiomas are WHO Grade 1 and indolent, a pattern of slow growth over serial future exams may establish meningioma as the presumed diagnosis, as in this case.

Although increased ^18^F-fluciclovine uptake in meningiomas has been documented in the literature, this case is unique for the following reasons. First, the patient’s history and potential risk factors include his age and previous metastasis to his iliac spine. Additionally, this patient has a known RB1 mutation, which has been implicated in facilitating prostate cancer metastasis [8].

## Conclusions

On ^18^F-fluciclovine PET/CT, meningiomas can mimic high-grade prostate meningeal metastases and are especially concerning when found in older patients in conjunction with previous bony metastases. Because meningiomas typically demonstrate indolent growth compared to meningeal metastases, prior imaging can differentiate between the two. Furthermore, an alanine peak at 1.47 ppm on spectroscopy is specific for meningiomas. For this patient with a known RB1 mutation, prior radiological studies established the diagnosis of meningioma and minimized the likelihood of meningeal metastasis from prostate cancer because of the tumor’s slow growth.
